# Photodynamic therapy of cervical cancer by eradication of cervical cancer cells and cervical cancer stem cells

**DOI:** 10.18632/oncotarget.27029

**Published:** 2019-07-09

**Authors:** Elvin Peter Chizenga, Rahul Chandran, Heidi Abrahamse

**Affiliations:** ^1^ Laser Research Centre, Faculty of Health Sciences, University of Johannesburg, Johannesburg, South Africa

**Keywords:** photodynamic therapy, cervical cancer, cancer stem cells, photosensitizer, cancer resistance

## Abstract

The heterogeneous nature of cancer puts cancer stem cells (CSCs) at the beating heart of the tumour. Because of their inherent characteristics of stemness, CSCs evade putative cancer therapies, resulting in treatment resistance or tumour recurrence after a seemingly successful treatment. To prevent treatment resistance and cancer recurrence, killing the beating heart of the tumour is of utmost importance. This study therefore, sought to determine the effect of Photodynamic Therapy (PDT) in eradicating cervical cancer and cervical CSCs. Cervical CSCs were isolated from a cervical adenocarcinoma cell line, HeLa cells, and grown in liquid medium incubated at 37° C, 5% CO_2_ and 85% humidity. Increasing doses of AlPcS_mix_ photosensitizer were administered to both the total cell population and the isolated CSCs, and irradiated using 673.2 nm diode laser. Post-irradiation cellular changes were observed using biochemical assays and microscopy to determine the response of both the total cell population and the CSCs. Results showed a dose-dependent response of both cell populations to treatment, by demonstration of significant morphologic changes, increased cytotoxicity, and decreased cell viability and proliferation. The study suggested that PDT using AlPcS_mix_ is a very effective treatment method for the eradication of cervical cancer cells and cervical CSCs, *in vitro*.

## INTRODUCTION

Increasing evidence supports the concept of tumour heterogeneity, which explains that like normal tissue, tumours comprise of cells at different levels of differentiation and maturity. The evidence shown in numerous studies has presented a subset of cancer initiating cells in cancerous tissue, commonly referred to as cancer stem cells (CSCs) which are responsible for tumorigenesis, metastasis, drug resistance, and recurrences [1]. Because they are biologically at different levels of maturity, the different types of cells in the tumour exhibit varying levels of sensitivity to genotoxic and cytotoxic drugs. Tumour heterogeneity therefore, forms the basis through which other cell populations resist treatment [2]. Due to the homogenous notion about cancer, chemotherapeutic drugs have not been very effective. Hence, the stem cell theory and the concept of tumour heterogeneity has provided insight into the development of novel therapeutic modalities that can successfully eradicate the cells of a tumour together with the more resistant stem cells at the apex.

Essentially, cells in the same tumour differ in virtually all phenotypic features [2]. Cancer cells at different levels of development, though arising from the same clone of cells, have variations in morphology, gene expression, metabolism, motility, and immunogenic and metastatic potential [3, 4]. Due to these inherent properties, cell purification studies have shown that CSCs are responsible for tumorigenesis, metastasis, drug resistance, and recurrences [5, 6]. Starting in 1994, the first report on isolation of CSCs from an acute myeloid leukaemia was documented by Lapidot *et al* [7]. Following this discovery, Bonnet and Dick [8] used cell purification studies to identify Cluster of Differentiation (CD) molecules in a subpopulation of leukemic cells which they reported as leukaemia initiating cells. To date, CSCs have been isolated in many solid tumours including cervical [9], breast [10], ovarian [11], melanoma [12], brain [13], pancreas [14], head and neck [15], and many others.

In the present day, CSCs have been well characterized and their role in treatment resistance, metastasis and cancer recurrence has been amply described. They are known for their enhanced drug efflux ability owing to the presence of membrane transporter proteins, the ABC family on their cell membranes [16]. Gene expression analysis of cervical CSCs correspondingly shows upregulation of cellular components responsible for DNA repair and the metabolism of Reactive Oxygen Species (ROS) in the cells [17]. Furthermore, CSCs contain a higher number of DNA repair proteins than the more mature cancer cells and they upregulate stem cell signalling pathways. They have slow cell kinetics and exist in hypoxic niches (Stem cell niche) which facilitate their escape from putative therapies including chemotherapy and radiation [17–19]. A rapid response to treatment after first line therapy is usually observed due to efficient killing of non-CSCs [20]. However, CSCs frequently survive, proliferate, and differentiate after therapy, resulting in tumour recurrence. This has led to much focus being directed towards the search for therapies that can effectively eradicate the CSCs at the core of the tumour to avoid cancer treatment failures and recurrences.

A long-standing treatment modality for that reason, Photodynamic therapy (PDT), has gained much attention, and it has extensively been studied and proven effective in treating cancer. PDT employs the use of a light excitable dye molecule called a photosensitizer, which selectively accumulates in tumour cells and induces cell death by generation of ROS and free radicals upon excitation by light of a particular wavelength [21]. In simple terms, PDT eradicates cancer cells by virtue of light and a PS which in the presence of molecular oxygen, yields a set of chemical reactions that generate ROS and other free radical species causing death by either or a combination of necrosis, apoptosis and autophagy [22]. *In vivo*, PDT also activates the host antitumor immune responses, and causes damage of the tumour vasculature, further enhancing the rate of cancer cell eradication [23, 24]. PDT has many advantages over other therapies. It is very specific with very few side effects, has little or no scarring effect after healing, it has lower costs and is tolerant to repeated doses [25]. Conventional treatments such as chemotherapy and radiotherapy conversely have been associated with increased side effects and limited efficacy. Studies have shown that PDT is a promising alternative approach for improved cancer treatment [25, 26].

When PDT is used to treat Cervical Intraepithelial Neoplasia (CIN) and cervical cancer, the side effects associated with cervical cancer therapies including pain and bleeding, are revoked. Unlike other therapies, PDT also preserves fertility, and has no negative effects on pregnancy and delivery [27]. Successful treatment with PDT however is extremely reliant on the choice of PS used. In cervical cancer treatment, there are two common PSs that have been used, including 5-aminolaevulinic acid (ALA) and Photofrin. ALA though not a photosensitizer in itself, has been used topically using a cervical cap and irradiated after 4–6 hours of drug application in PDT of CINs. It has shown significant regression of CIN lesions, but without entire eradication of the lesion [28, 29]. Photofrin on the other hand, was proven effective in more than 80% for CIN I and CIN II, over 90% for CIN III, with a remarkable 100% remission in squamous cell carcinoma lesions [30]. However, its use is associated with skin irritation and photosensitivity after treatment, including episodes of optic hyperesthesia and oedema in other patients [27, 30]. Phthalocyanines alternatively, have shown great potential for use in PDT due to their high ROS producing ability, strong absorption in the tissue-absorption wavelength range and high tumour uptake levels. Among many other metallised phthalocyanines used in PDT, Aluminium Phthalocyanines have proven to be good photosensitizing compounds for many solid tumours including breast, colon, oesophageal and many others. This study therefore, reports the effectiveness of PDT using AlPcS_mix_ as a photosensitizer for the eradication of cervical cancer cells, and cervical CSCs, *in vitro*.

## RESULTS

### Fluorescence spectra of AlPcS_mix_

**Figure 1:**
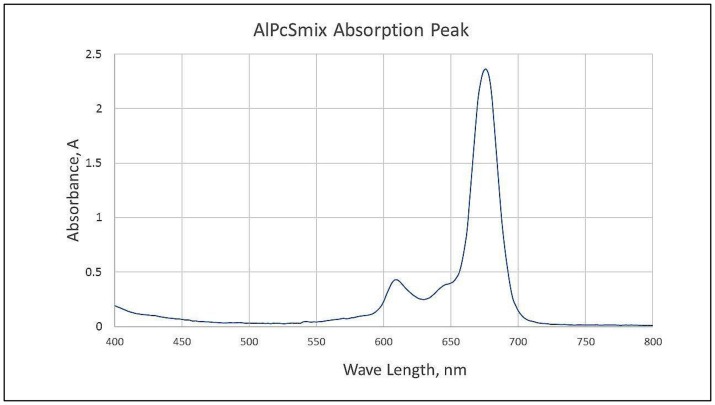
Spectrophotometric analysis of AlPcS_mix_ showing the maximum absorption/excitation peak at 674 nm.

Fluorescence spectra of AlPcS_mix_ was determined using UV-Vis Spectrophotometry, and results indicated maximum absorption at 674 nm when measured at 2 nm wavelength intervals from 400 nm to 800 nm. In Figure 1, the wavelength at which AlPcS_mix_ has the highest absorption is indicated and it presents the peak at which the PS gets excited. The spectral range of 656 to 692 however, is tolerable for activation of the PS. Presented in Figure 1, is a typical minor groove showing characteristic splitting of the Q band at ± 610 nm region at pH 7.4. The increased absorption in this region is simply due to the formation of aggregates, a common phenomenon in differently sulfonated phthalocyanines at neutral pH. We used a 673.2 nm semiconductor diode laser in this study to activate the PS.

### HeLa side population express CD133 and CD49f surface markers

**Figure 2:**
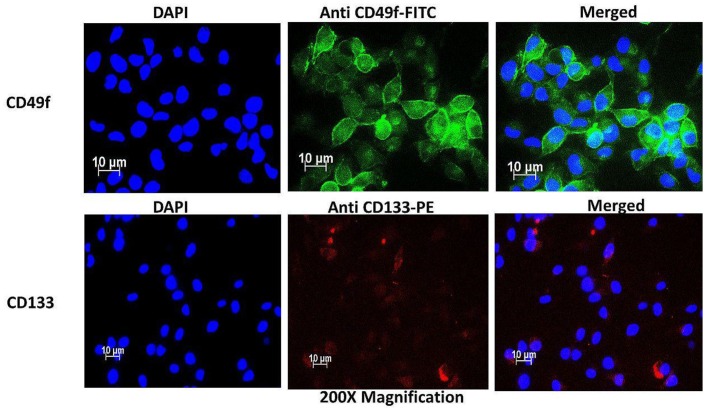
Immunofluorescence characterization of cervical side population cells indicating the presence of cervical CSC surface markers CD49f stained with FITC (green) and CD133 stained with PE (Red).

**Figure 3:**
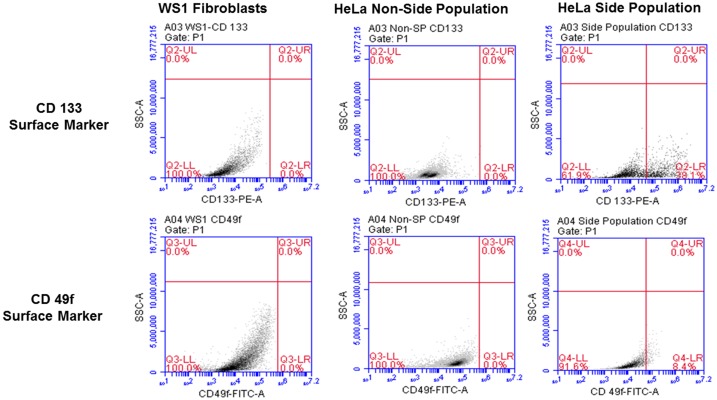
Flow cytometric analysis of cervical side population cells showing surface markers with 38.1% positive for CD133 and 8.4% positive of CD49f. The non-side population as well as the negative control, WS1 fibroblast were negative for both markers.

The side population isolated from the total cells was 0.7% on average, using magnetic-bead separation. Immunofluorescence characterization of these cells showed positive signals of stem cell surface marker CD133 and cervical CSC surface marker CD49f. Direct staining of the side population using PE-conjugated anti-CD133 indicated the presence of CD133 surface antigen (Figure 2). Likewise, FITC-conjugated anti-CD49f showed positive expression in the side population. These results indicated that the separated side population was indeed CSCs of cervical tissue. To further confirm the presence of these markers, flow cytometric analysis was performed. The results showed that 38.1% of the side population were positive for the surface marker CD133 and 8.4% were positive for CD49f. This confirmed positive isolation of cervical CSCs, which in the sorting of cells positively expressing CD 133, the cells also confirmed expression of CD 49f (Figure 3).

### Cervical cancer side population cells possess a high Hoechst efflux ability

**Figure 4:**
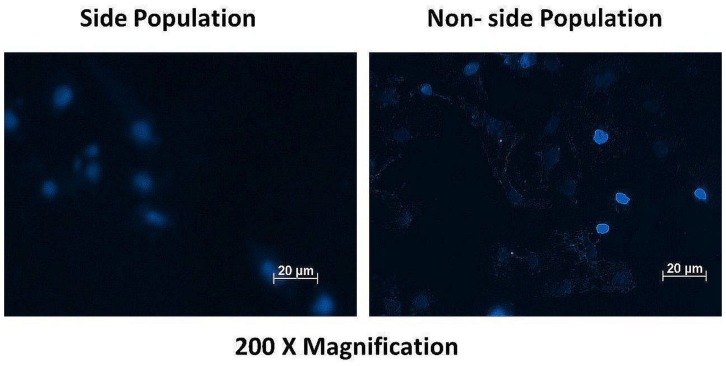
Fluorescence microscopy of the side and non-side population after staining with the dye Hoechst 33342 showing high fluorescence intensity (+++) in the non-side population of cells and a low fluorescence intensity (+) in the side population cells.

Both the side and non-side population cells were stained with Hoechst dye to qualitatively determine the concentration of the dye in the cells. The fluorescence intensity of the dye in the two populations were notably different when detected using the DAPI filter at 200× magnification. The non-side population showed high fluorescence intensity (+++) compared to the side population showing low fluorescence intensity (+), indicative of CSC’s high Hoechst efflux ability. Figure 4 shows the fluorescence signal of both cell populations.

### Fluorescence analysis indicates the accumulation of AlPcS_mix_ inside cervical cancer cells and cervical CSCs

**Figure 5:**
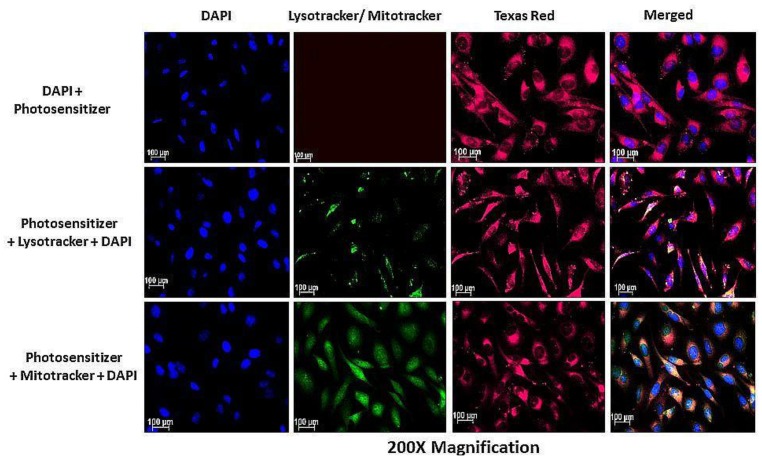
Subcellular localization of AlPcS_mix_ in HeLa cells showing the localization of the PS (Texas Red) in the cytoplasm (red), lysosomes and mitochondria (seen as blending of green and red).

**Figure 6:**
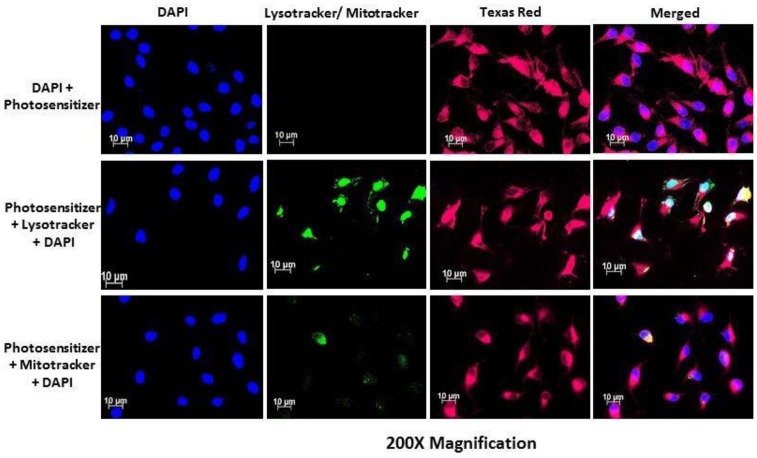
PS subcellular localization in HeLa side population indicating accumulation of the PS (Texas Red) in the cytoplasm of the HeLa side population. Mitotracker and lysotracker (green) showed no significant localization of the PS in the mitochondria and lysosomes.

Subcellular localization of AlPcS_*mix*_ was determined using fluorescence microscopy, which demonstrated substantial PS uptake by both the total cell population and the side population, with most of the PS accumulating in the cytoplasm of the cells. Figure 5 shows auto fluorescence of AlPcS_*mix*_ in Texas red and intracellular organelles, mitochondria and lysosomes in green. AlPcS_mix_ was shown to accumulate in the cytoplasm of HeLa cells with localization in both mitochondria and lysosomes. Figure 5 shows the merged orange colour due to accumulation of the PS in these organelles. The side population cells also showed accumulation of AlPcS_mix_ in their cytoplasm but unlike the total cell population, there was diminutive localization of the PS in the mitochondria and lysosomes (Figure 6). This difference has an important influence on the response of these cells to treatment as shown in preceding sections.

### Photodynamic therapy causes sufficient damage to both cervical cancer mature cells and cervical cancer stem cells

#### Morphology

**Figure 7:**
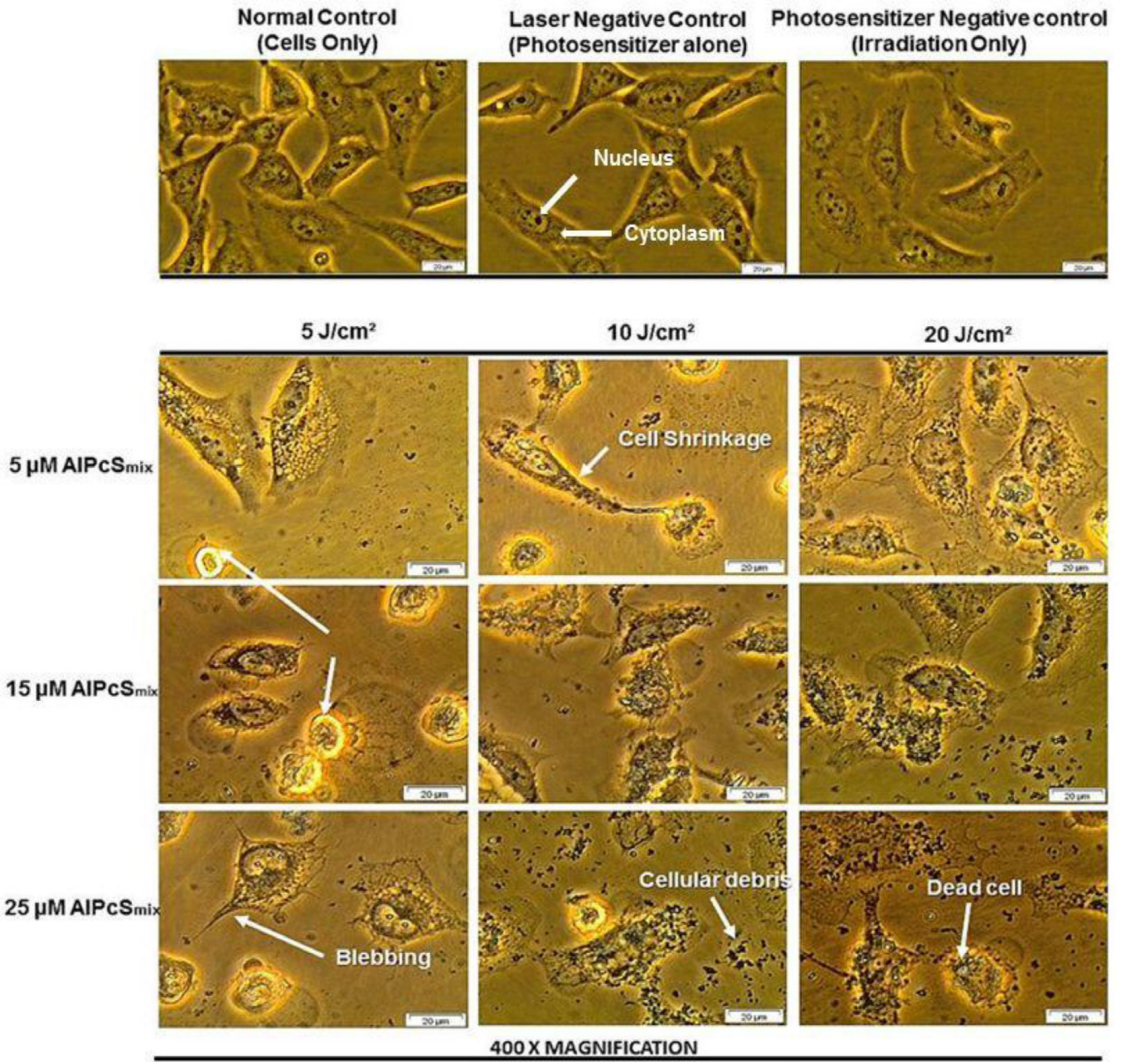
Cellular morphology of HeLa total population as seen under 400× magnification demonstrating unaltered structure of cells in the control group and groups treated with either of the variables alone and notable distortion in structure in PDT treated cells.

**Figure 8:**
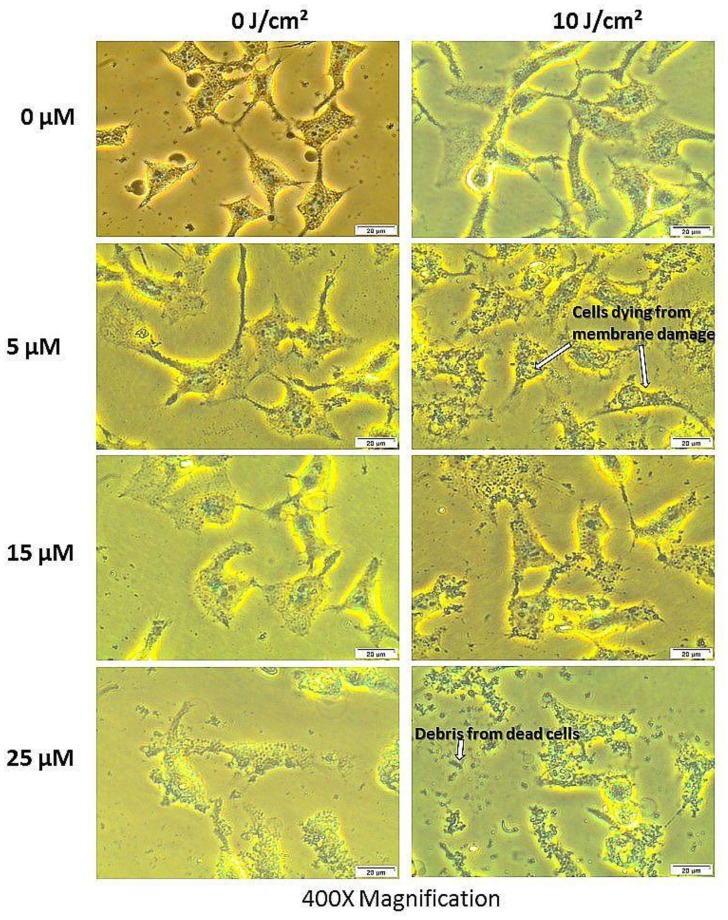
Cellular morphology of the side population showing unaltered structure of cells not treated with either of the variables alone and notable structural changes in PDT treated cells as seen using 400× magnification.

PDT treated cells and control groups were examined for morphological alterations after 24 hours of post irradiation incubation using 400× magnification as shown in Figure 7. Figure 7 shows the morphology of the total cell population and Figure 8 shows the morphologic assessment of the side population cells. As seen in the diagrams, cells in control group 1 that comprised of cells neither treated with AlPcS_mix_ nor exposed to light appeared structurally unaltered and retained their characteristic morphology after 24 hours of incubation. Similar results were demonstrated in cells that received either PS alone without irradiation (control group 2) or those that received irradiation alone without PS (control group 3). The cells appeared healthy and uninjured and actively proliferated to confluence in 24 hours. PDT treated cells on the other hand (AlPcS_mix_+ irradiation) showed visible structural alteration with increasing level of damage proportional to the increase in PDT doses. Although there was significant cell damage in both cell populations, the side population incurred less damage than the total cell population at similar dose concentrations. There was however observable cell morphology alterations showing cell shrinkage, blebbing and detachment from the plate surface.

#### Viability

**Figure 9:**
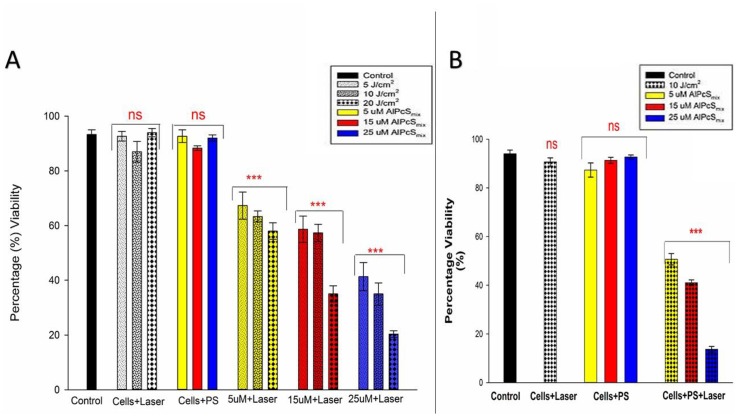
Post-irradiation viability. (**A**) Total population cell viability showing a dose dependent decrease in cell survival of PDT treated cells (*p* < 0.001) after 24 h incubation with insignificant decrease (ns) in control group and groups treated with either of the variables alone. (**B**) Side population cellular viability indicating significant decrease in cell viability (*p <* 0.001) in PDT treated cells after 24 h with insignificant decrease (ns) in control cells and those that were only treated with either of the variables alone.

Figure 9 shows percentage viability that represents the effect of PDT on the survival of the cells after treatment. As seen on the graph, the control cells were viable after 24 hours of incubation with a viability of over 95%. The PDT treated cells conversely, showed a dose dependent decrease in viability with a much reduced percentage seen in cells that received the highest dose of treatment. The untreated cells with PS and irradiation alone showed no significant decrease in viability, indicating that neither PS nor irradiation can induce any effect separately. The results therefore demonstrate active reduction in viability as a result of PDT. In Figure 9A, the total cell population shows a dose dependent decrease in viability and Figure 9B shows the different percentages of viable and nonviable side population cells after treatment.

#### Cytotoxicity

**Figure 10:**
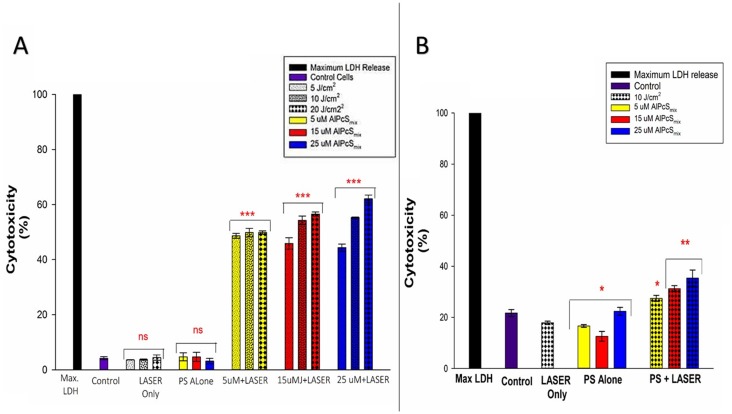
Post-irradiation cytotoxicity. (**A**) Total population cytotoxicity demonstrating the release of LDH. PDT treated cells showed a significant increase in LDH (*p *<0.001) in all PDT groups with a dose dependent increase in the level of cytotoxicity after 24 h with no significant increase in LDH (ns) in control group and groups treated with either of the variables alone. (**B**) Side population cytotoxicity in PDT treated cells indicating a mild significant increase in LDH release in PDT treated cells that received 5 μM of AlPcS_mix_ (*p *< 0.05), and a significant value of (*p *<0.01) in cells that received 15 and 25 μM of AlPcS_mix_ after 24 h of incubation.

Cell membrane damage was demonstrated by detection of the level of LDH enzyme released into the culture media after 9 hours of post-irradiation incubation. High levels of LDH were observed in PDT treated cells whose cell membranes ruptured as a result of PDT toxicity on the cells. As seen in Figure 10A and 10B, the control cells in both populations showed no significant LDH release compared to the PDT treated cells. The results demonstrate that PDT induces cells lysis by processes that ultimately alter cell membrane integrity causing cell death. As observed in the morphology, the extent of cell damage however was different between the two populations with similar doses. The side population cells seemed to have less damage when compared with the total cell population at the same dose. Though side population cells were not very susceptible to damage and required a higher dose, it was demonstrated still, in both populations that cell damage increased with increasing doses of PDT.

#### Proliferation

**Figure 11:**
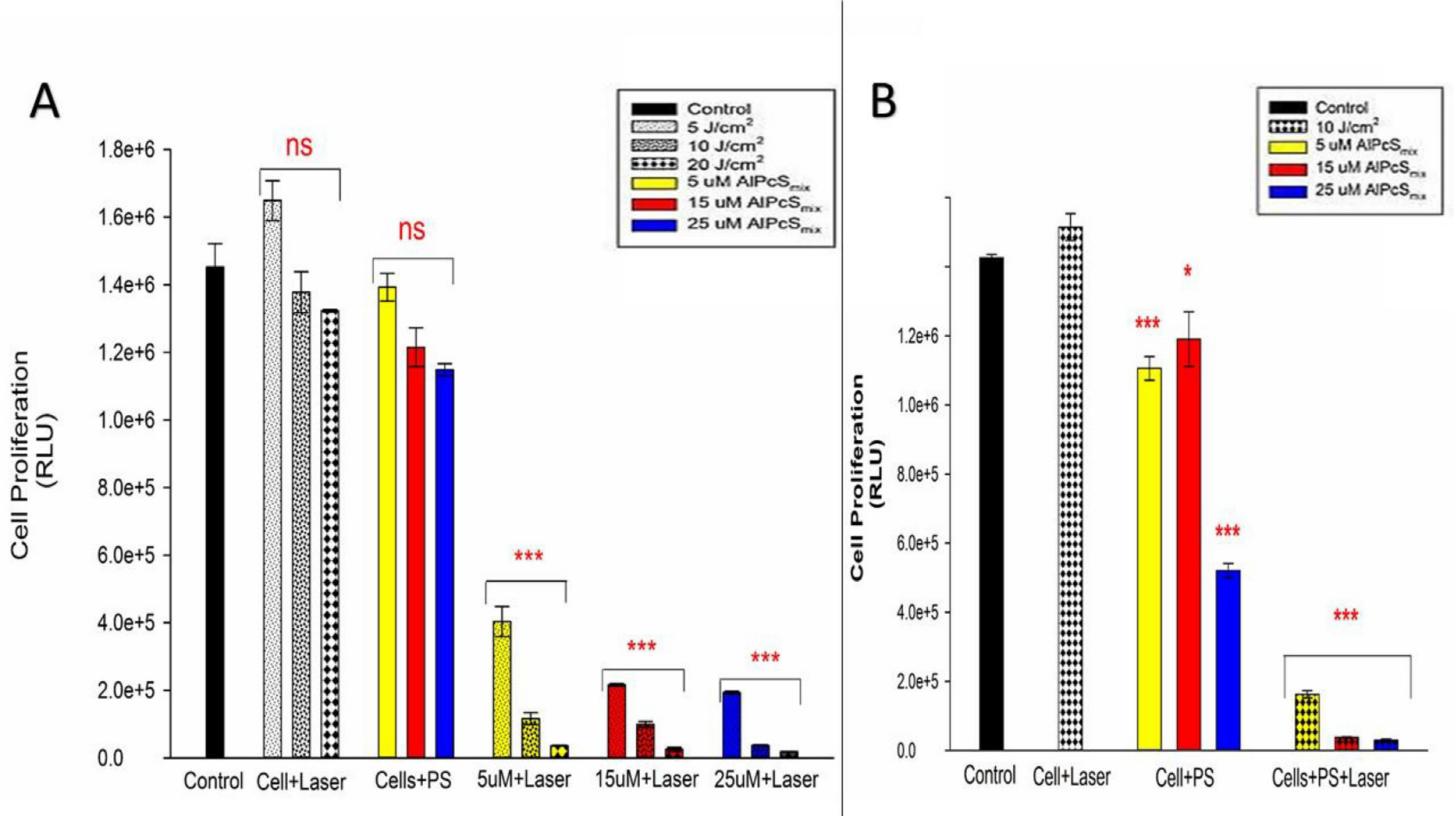
Post-irradiation cellular proliferation. (**A**) Total population ATP luminescent signal indicating significant decrease in proliferation of PDT treated cells (*p *< 0.001) after 24 h and insignificant decrease in cell proliferation of the control cells and groups treated with either of the variable alone. (**B**) Side population luminescent signal showing significant decrease in cell proliferation (*p * <0.001) after 24 h. Cells treated with PS alone also showed significant decrease in cell proliferation (*p *< 0.05 and *p*< 0.001) nevertheless considerably minimal compared to the PDT treated cells.

There was a substantial decrease in cell proliferation in PDT treated cells compared to the control and untreated cells. As shown in Figure 11, the control group showed a high proliferation rate in 24 hours. Cells that received PS without irradiation and those that received irradiation alone did not show significant decrease in Adenosine Triphosphate (ATP) production indicating that neither of the conditions alone have any effect on the cells proliferation. The PDT treated cells on the other hand showed a decrease in ATP production in a dose dependent manner with the highest decrease observed in cells that were treated with 25 μM of AlPcS_mix_. These results indicate that treatment with PDT impaired the cells ability to grow and proliferate, resulting in cell death. A noteworthy observation was made in control cells that were irradiated without addition of AlPcS_mix._ These cells in both populations demonstrated the ability of light alone to increase the proliferation of cells. This is a very important feature of light that has a significant implication in the process of treatment. As seen in the Figure 11, cells irradiated in the absence of AlPcS_mix_ proliferated to numbers exceeding the normal control group.

### Normal cell population using WS1 fibroblasts demonstrate mild susceptibility to photodynamic therapy

**Figure 12:**
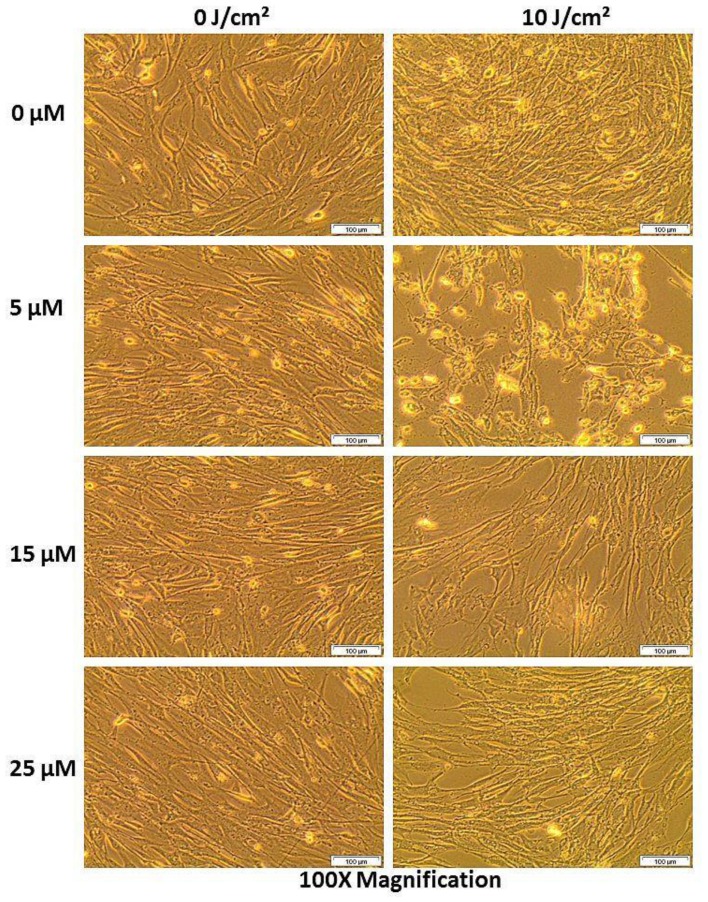
Post-irradiation morphologic assessment of WS1 fibroblasts indicating structural changes in normal cell population after 24 h of incubation (different concentrations aligned in vertical position and the fluences in horizontal position). There was no notable morphologic changes in all groups except for the PDT treated cells at 5 μM of PS, which showed notable cell rounding and detachment.

**Figure 13:**
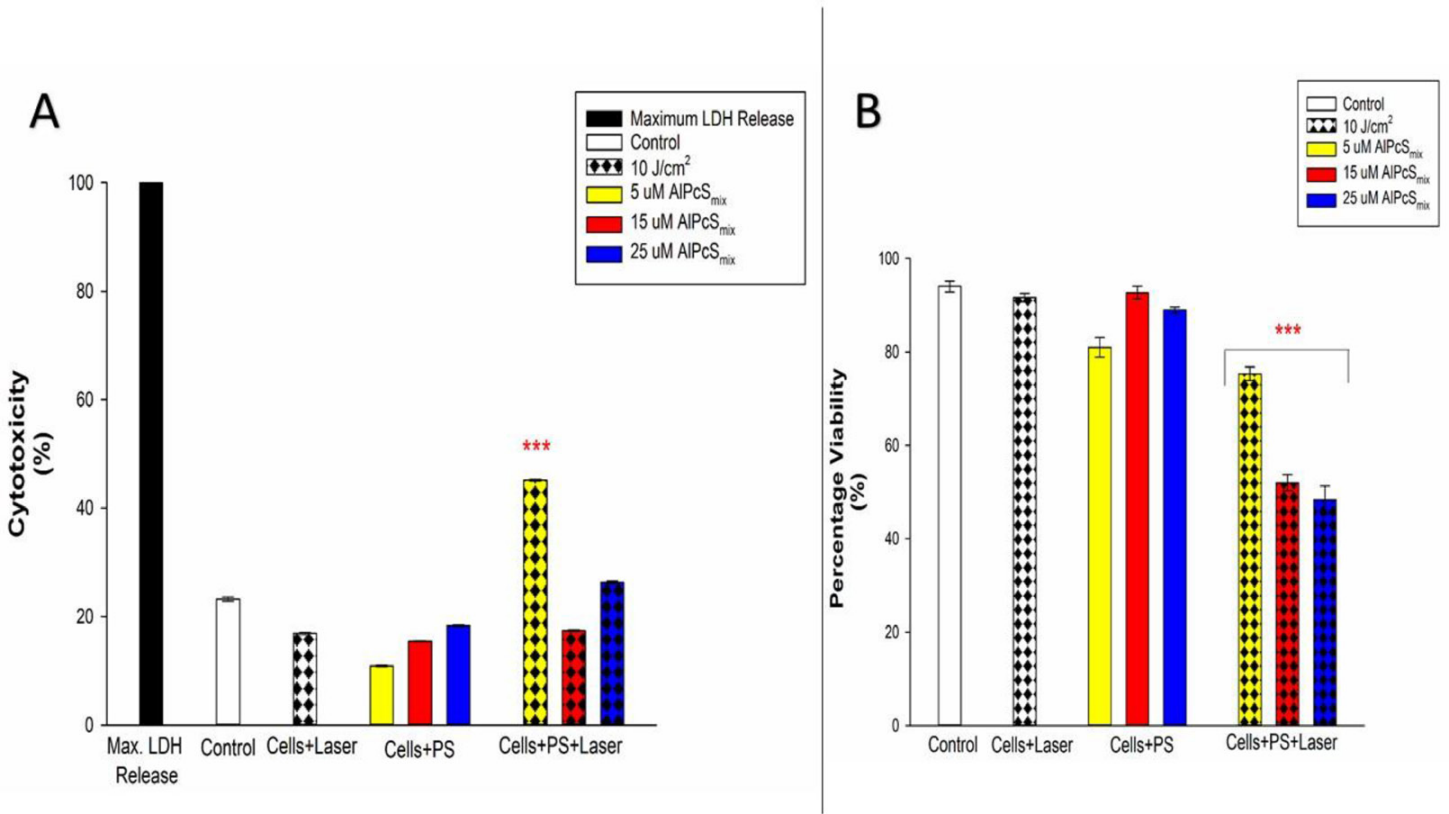
Post-irradiation biochemical response of normal cells exposed to PDT. (**A**) Cytotoxicity of treated WS1 fibroblasts indicating insignificant increase in cytotoxicity of both treated and untreated cells with the exception of cells that received 5μM of PS which showed significant increase in cytotoxicity (*p *< 0.001). (**B**) Viability of treated WS1 fibroblasts showing a significant dose dependent decrease in the viability of PDT treated cells (*p *< 0.001) after 24 h of incubation. Control cells and groups that received either of the variables alone did not show significant decrease.

To check the effect of PDT on normal cell populations surrounding tumors, WS1 fibroblast were used as a model. These cells upon treatment with PDT showed a mild but significant cellular damage, post-irradiation. Unpredictably with the WS1 cells, cellular responses were not dose dependent. Cellular morphology assessment showed slight detachment of the cells from the bottom of the culture dish in the group that was treated with 5 μM of PS and 10 J/cm^2^. Higher concentration did not show obvious changes in structure after 24 h of post-irradiation incubation (Figure 12). LDH cytotoxicity assay also showed similar response with insignificant release of LDH in higher concentrations and a mild increase in cells treated with 5 μM of PS. Viability of all groups were above 50% indicating lower number of dead cells in proportion to the total. Figure 13A shows the LDH released from the cells after PDT and Figure 13B show the percentage viability of the cells.

## DISCUSSION

Cervical cancer recurrent rates are overwhelming. An overall recurrence rate of over 30–50% of all treated cases has been reported along the years [31]. Many therapeutic interventions including surgical and chemotherapeutic drugs have been studied and implemented but to date, there is still a high mortality rate due to cancer resistance and recurrent disease. Putative treatment modalities have shown limited therapeutic potential with concomitant side effects, poor prognosis, and high rates of cancer relapse with a general reduced quality of life [32]. Enough evidence has pointed out to the presence of CSCs which resist therapy and drive tumour metastasis and recurrence. Chemotherapy and radiotherapy frequently kill bulk tumour cells while leaving these CSCs thriving and therefore augment their survival and proliferation, causing unfavourable outcomes in cancer treatment. In this present study we therefore assessed and demonstrated the effectiveness of an alternative therapeutic method, PDT, which can potentially drive down the hurdles in treating cervical cancer.

We used AlPcS_mix_ and a semiconductor diode laser of wavelength 673.2 to induce cancer cell damage *in vitro*. The absorption peak of AlPcS_mix_ was determined at 674 nm, wavelength at which the PS has its maximum absorption/excitation. A minor groove seen at ±610 nm was seen, indicating the typical splitting of the Q band at 610 nm at pH 7.4 which is characteristic feature of sulfonated phthalocyanines that results from the formation of aggregates [33, 34]. The spectrophotometric analysis showed that the range of 656 to 692 nm is tolerable for activation. In a study by Kresfelder *et al* (2009), AlPcS_mix_ was activated using a wavelength of 680 nm and they reported significant PDT results on an oesophageal cancer cell line [35]. In this study, excellent activation of AlPcS_mix_ was demonstrated in the post-irradiation cellular changes.

Before exposing cells to laser light, assessment of the subcellular localization of the AlPcS_mix_ using immunofluorescence showed increased entry and localization of the PS into the cells. Studies have shown that localization of the PS in specific sites of the cell, determines the type of cell death to be induced [22]. Depending on where the PS localizes, the damage may occur on plasma cell membranes or on intracellular organelles and their membranes. In PDT therefore, the cancerous cells are killed by different processes including necrosis, apoptosis, autophagy, and *in vivo*, by activation of the host antitumor immune responses, and damage of the tumour vasculature [22, 23]. Cytoplasmic accumulation and localization in cytoplasmic organelles has been shown to cause cell death by activation of apoptosis. Destruction of the vascular tissue and cell membranes causes a necrotic death. In this present study, it was shown that AlPcS_mix_ accumulated in the cytoplasm of the total cell population along with adequate localization in the mitochondria and lysosomes. In the side population, AlPcS_mix_ accumulated in the cytoplasm but unlike the total cell population, the drug did not show noteworthy localization in the mitochondria and lysosomes. This observed case is most likely due to the presence of higher amounts of ABC transporters on organelle membranes than the plasma membrane.

Normally, specific ABC transporters are found on plasma membrane and organelle membranes for active transport of molecules into and out of the cytoplasm. On CSCs, these proteins are overexpressed and play a major role in the metabolism of the cells and the transport of molecules, including the efflux of drugs in this case. We therefore postulated based on the observed outcome that these side population cells have a higher expression of similar transporters on their organelles that are responsible for effluxing the AlPcS_mix_ that entered organelles, back into the cytoplasm. Of the 48 human ABC genes characterized, only three main ABC transporters have been implicated in multidrug resistance of cancer cells [36]. These three main ABC transporters include, MDR1 (also called P-glycoprotein, ABCB1), MRP1 (also called multidrug resistance protein 1, ABCC1) and the ABCG2 multidrug transporter (also called BCRP/ MXR). Though other transporters have been implicated, the multidrug resistance phenotype in cancer cells is primarily a result of the overexpression pattern of these proteins.

Similar proteins have been demonstrated in cervical cancers. A recent finding has shown that in cervical cancer, MDR1 gene expression is associated with poor patient survival [37]. This observation strongly supports the presence of ABC transporters in cervical cancers which based on previous discussions, are most probably associated with the CSC population of the tumour. The ABCG2 protein has also been found in other cervical cancer cell lines and its presence in side population cells was associated with colony forming efficiency and the capacity to proliferate producing more side population cells and differentiation to more mature cancer cells [38]. Another protein, Bcl-X found in cervical side population confers resistance of cervical cancer to common chemotherapeutic drugs including cisplatin and doxorubicin, and also radiotherapy using γ-radiation [39]. Other numerous markers including high aldehyde dehydrogenase (ALDH) activity and the molecule called Brother of the regulator of the imprinted site (BORIS) variant subfamily 6 have been associated with cervical CSCs to confer resistance of cervical cancer [40, 41]. Nevertheless, because of the high affinity of PSs to cancer cells, there was significant accumulation of the PS in the cytoplasm which upon laser irradiation, enough ROS was produced to cause significant cell damage to the cells.

The PDT response of the side population was shown to be lesser compared to the total cell population. The cellular responses of both populations however showed that, the process of PDT was the core source of the observed phenotypic changes and cytotoxicity. Neither of the individual components of PDT alone induced any observable phenotypic alterations. The experiments were set up to show effect of each individual component of PDT on the cells. A control group comprising of cells not treated with either PS or light confirmed the sustained viability and proliferation. Control groups comprising of either PS or light alone did not show any significant phenotypic alterations and were in like manner very viable and proliferated in culture. Contrary, cells which received different concentrations of AlPcS_mix_ and exposed to irradiation demonstrated affirmative features of cytotoxicity and impaired proliferation. Morphologic changes using bright field microscopy showed marked structural changes that represent cells undergoing senescence. Cell death features i.e. blebbing and shrinkage were noticeable using 100× magnification.

ROS and other reactive free radical species are produced by the PS in its activated state which directly cause cell death by interactions with cellular components and oxidization of biomolecules. Cell membrane damage was determined using the LDH cytotoxicity test which showed a high LDH release into the cytoplasm in PDT treated cells in a dose responsive manner for both cell populations. The outcome presented that PDT induced sufficient reactions that damaged the plasma membrane. The control groups did not show significant increase in LDH. To further demonstrate cell damage, ATP proliferation and trypan blue viability assays significantly presented a decrease in cellular proliferation and viability respectively, in PDT treated cells. After 24 hours, PDT treated cells lost their function and failed to proliferate in culture having given sufficient nutrients and optimum culture conditions.

AlPcS_mix_ has shown desired therapeutic effects in many solid tumours in previous studies [42, 34, 35]. Zharkova *et al* (1995) used AlPcS_mix_ to treat patients with various types of cancer, *in vivo*, from which the majority showed complete regression of the tumours [42]. In another study by Kresfelder *et al* (2009), it was shown that AlPcS_mix_ induced sufficient cell death in oesophageal cancer cells with significant alteration of the post-irradiation cell proliferation [35]. It was also demonstrated in the same study that AlPcS_mix_ had the most prominent effect when compared to a different PS, GePcS_mix_. In cervical cancer cells, PDT using a different metallised phthalocyanine, ZnPc has proven effective in inducing cell damage through activation of apoptosis, necrosis and autophagy [43, 44]. Our results demonstrated a similar effect of AlPcS_mix_ in the eradication of cervical cancer HeLa cells. However, we observed two phenomena that may cause unfavourable outcomes if PDT is not amply optimized prior to therapy, including potential photobiomodulation of tumour cells and cytotoxicity of normal cells.

Our results showed enhanced proliferation of cells that were irradiated with 5 J/cm^2^ without addition of PS. These cells grew in number exceeding the total number of the control cells that were not exposed to either light or PS treatment. This observation demonstrates that 673.2 nm wavelength induced the proliferation of the cells. Existing data verifies that depending on the type of cell, exposure to different wavelengths at different fluences can either stimulate or inhibit the proliferation of cells [45, 46]. Crous and Abrahamse (2016) demonstrated that lung cancer cells were stimulated at the wavelength range of 636 nm with lower fluences [46]. Nonetheless, we showed that proliferation was immensely inhibited in the presence of the PS. This observation proposes the risk of cancer impulsive propagation in the case of exposing light to cells that have not actively absorbed the PS. It is therefore utterly important to ensure and confirm sufficient PS absorption and localization before irradiating cells with light.

Because tumours are surrounded by other noncancerous tissue cells that are not freed from PDT exposure, we used a control group to represent the normal pool of cells in the body. For proper tissue regeneration after cancer eradication, normal cells of the tissue and connective tissue cells need to be preserved. WS1 fibroblasts were used to check the effect of PDT on the non-cancerous cells around the tumour. The response of these cells at varying PDT doses showed a very slight but significant alteration in morphology, viability and increased cytotoxicity. This demonstrates that normal cells do take up the PS though at a very slow rate. It is renowned that different cells have diverse PS uptake kinetics i.e. a distinct ratio of photosensitizer uptake and clearance [47]. Tumour cells have a high affinity for PSs due to the increased permeability and overexpression of membrane transport molecules while normal cells have a very low affinity due their highly controlled transport of molecules. Increased number of LDL receptors on tumour cells increases PS uptake via endocytosis of LDL-PS complexes [48]. Normal cells on the other hand have very few LDL receptors on their surfaces and a highly regulated movement of molecules through their membranes. On long-term exposure to circulating PS, sufficient time is allowed for the PS to move into the cells.

Castano *et al*., (2004) reported that PDT could cause excessive tissue destruction as observed in this example when enough PS enters normal cells [49]. This can be taken care of by optimizing the overall exposure time of the PS before irradiation. Accumulation of the PS in cancer cells is utterly dependent on factors including overall time of exposure and the concentration of the PS [50]. Incubation period of the PS prior to light exposure is therefore a very important factor to consider in PDT. Fortunately, there are other factors that enhance affinity of the PS to tumour cells *in vivo,* including the acidic intracellular pH, high collagen content, leaky microvasculature and the poor lymphatic drainage in tumour tissue [50]. Because of these factors and the increased number of LDL receptors on tumour cells, PDT will result in extensive tumour tissue destruction and minimal normal cell injury and hence the need for thorough uptake optimization studies prior to PDT, in order to maximize the former and avoid the latter.

In conclusion, we suggest and recommend the use of PDT for treatment of cervical cancer. PDT using AlPcS_mix_ has proven effective in eradicating both the bulk tumour cells and the more resistant CSCs of the tumour, *in vitro*. Observable phenotypic changes in cells with significant decrease in proliferation and increased cytotoxicity were demonstrated. Nevertheless, preferred therapeutic potency is highly dependent upon a well assessed dose response study devising the highest cancer inhibitory concentration while ensuring the lowest possible effect on the normal cell population.

As observed in this study, though PDT is known for its selectivity and high affinity for cancer cells, higher doses and long PS exposure time, do have a significant effect on the normal cell population and therefore a dose response should always be amply optimized to increase treatment efficacy while reducing normal cell injury. Future studies should also consider checking this effect on normal cervical cells to adequately determine the specificity of this PS in treating cervical cancer. Previous research has also shown possibility of specific active targeting of cancer cells and CSCs using monoclonal antibodies conjugated to PS to avoid toxicity to normal cells and the use of nanoparticles to increase bioavailability and PS uptake. These ideas should be considered in PDT of cervical cancer. Lastly, further demonstration of the effects of PDT on CSCs in a 3D model using tumour spheroids and *in vivo* experimentation is recommended in order to demonstrate the true extent of PDT for treating cervical cancer in a clinical setting.

## MATERIALS AND METHODS

### Cell culture

Cervical cancer, HeLa cells (adenocarcinoma, HPV18, ATCC^®^ CCL2™) were directly procured from ATCC (American Type Culture Collection, Manassas, VA, USA). These cells were amply characterized and authenticated by the ATCC using PCR for viral genome sequencing and DNA profiling, cytogenetic analysis was also performed by the ATCC for a full description of the cytogenetic information of the HeLa cells. The cells were passaged at the Laser Research Centre, University of Johannesburg upon receipt following ATCC’s recommendations for thawing frozen vials. They were cultured in liquid medium, Minimum Essential Medium Eagle’s, MEME, (Sigma Aldrich: M2279). Complete media was prepared by supplementing MEME with 10% Foetal Bovine Serum, FBS, (Sigma Aldrich: F0804), 1% sodium pyruvate (), 2% L-glutamine (), 100 mg penicillin and streptomycin (Sigma Aldrich: P4333100ML), and 100 mg Amphotericin B (Sigma Aldrich: A2942-100ML). The cells were cultured as a monolayer in an incubator set at 37° C with 5% CO_2_ and 85% humidity.

### Cancer stem cell isolation and characterization

Cervical CSCs, also referred to as side population were isolated from cultured HeLa cells using Magnetic Activated Cell Sorting, MACS (Miltenyibiotec). CD133 surface marker was used to select CD133 positive side population from the CD133 negative non-side population. The CD133 MicroBead Kit is a magnetic labeling system designed for the positive selection of CD133 positive cells. In this study, the QuadroMACS™ Separator (Miltenyibiotec) was used for positive selection of HeLa side population. A single cell suspension was prepared from cultured HeLa cells and placed in the QaudroMACS™ Separator which magnetically separates the labelled CD133 positive cells while depleting the CD133 negative non-side population. The CD133 negative cell fraction was corrected in MACS collection tubes as the non-side population. Separated cells were cultured in Dulbecco’s Minimum Essential Medium F12, DMEM-F12 (Sigma Aldrich) supplemented with 0.5% FBS, 10 ng TGT, 20 ng EGF, 100 mg penicillin and streptomycin (Sigma Aldrich: P4333100ML), and 100 mg Amphotericin (Sigma Aldrich: A2942-100ML), in utra-low attachment flasks. Immunofluorescence and flow cytometric analysis of the side population was performed using PE conjugated anti-CD 133 antibodies (BD/566593 Hu CD133 PE W6B3C1) and FITC conjugated anti-CD 49f antibodies (BD/561893 CD49f FITC MAB), to characterize the side population. Hoechst staining (Hoechst 33342 Invitrogen) was also used to further characterize the side population.

### Photosensitizer preparation and fluorescence determination

a stock solution of 0.01 M Sulfonated Aluminium Phthalocyanine Mix, (AlPcS_mix_) also known as photosens, was prepared from solid form AlPcS_mix_ of molecular weight 845 g/mol (Department of Physics, Rhodes University, RSA). Spectrophotometric analysis of the PS at pH 7.4 in phosphate buffered saline (PBS), was done using the Genway UV-Vis Spectrophotometer (Lasec) set at 2 nm wavelength intervals from 400 nm to 800 nm. Fluorescence spectra and absorbance were then determined and plotted on a dot graph to determine the maximum absorption/excitation wavelength.

### Cell seeding and addition of photosensitizer

PDT experiments were performed successively starting with the total cell population, and based on the outcome, the investigation proceeded to PDT of the side population and lastly the WS1 fibroblast that represented the normal pool of cells around the tumor. All cell types were seeded into 3.4 cm^2^ culture plates (Corning Inc.) at a density of 3 × 10^5^ cells per plate and incubated for 8 hours in supplemented growth medium, DMEM-F12 for side population and MEME for total population and WS1 fibroblasts to allow attachment. Varying concentrations (5 μM, 15 μM and 25 μM) of AlPcS_mix_ were added to the plates and incubated in the dark at 37° C with 5% CO_2_ and 85% humidity for 12 hours. After 12 hours, media was removed from the plates and the cells were rinsed 3 times with pre-warmed Hanks Balanced Salt Solution, HBSS (Sigma) to remove all traces of floating PS.

### Localization

A qualitative analysis of the PS uptake and localization was performed using fluorescence microscopy to detect the red auto-fluorescence of AlPcS_mix_ in the cells. Cells were grown on glass cover slips in 3.4 cm^2^ cell culture plates. The cells were incubated as described previously to allow attachment and were then treated with 5 μM of AlPcS_mix_ and re-incubated for 12 hours to allow maximum absorption. After 12 hours the cells were washed 3 times with HBSS and stained with pre-warmed probe containing medium with 100 nM mitotracker (Invitrogen M7514) and 65 nM lysotracker (Invitrogen) separately and DAPI was used to counterstain. An unstained control and a negative control (stained cells without PS, with DAPI counterstain) were included. Images were captured using 20× objective on a Carl Zeiss Axio Z1 microscope (Carl Zeiss MicroImaging GmbH), with 490Ex/516Em filters for mitotracker, 380Ex/576Em for lysotracker, 359Ex/461Em for DAPI and 649Ex/670Em for AlPcS_mix._

### Laser irradiation

PDT experiments were conducted to determine the response of the cells at different doses. First, the total cell population that was treated with different concentrations of AlPcS_mix_ was irradiated using a 673.2 nm diode laser (Oriel, USA) at fluences of 5, 10 and 20 J/cm^2^. Appropriate controls were included to check the effect of each individual variable separately. A negative control comprising of cells only, without PS and irradiation was included to rule out any changes in cell biology caused by factors other than the variables of this study. Laser negative controls comprising of cells that received the different concentrations of PS without irradiation checked for any possible effects of the PS in its inactive form and lastly a PS negative control comprising of cells that were exposed to light without addition of AlPcS_mix_ checked the effect of light in the absence of the PS. Exposure times for the different fluences were calculated from the power output in mW which was measured using the coherent Fieldmate detector and sensor. During irradiation, all groups including controls were left in 1 mL of media in the incubator and were only taken out during the time of irradiation to reduce effects of nuisance variables on cell growth i.e. prolonged exposure to room temperature, gases and humidity, during the experimental process. After irradiation, all plates were re-incubated at 37° C with 5% CO_2_ and 85% humidity. The same was done with the side population and the normal cell population (WS1 fibroblasts), however using only 10 J/cm^2^ as it was observed that 10 J/cm^2^ was sufficient to activate the PS.

### Cell cytotoxicity

Cellular cytotoxicity was determined using Lactate Dehydrogenase (LDH) assay, CytoTox 96^**®**^ Non-Radioactive Cytotoxicity Assay (Anatech: Promega, PRG1780). LDH is an intracellular enzyme that is released into the extracellular space upon cell lysis. The enzyme catalyses the conversion of lactate to pyruvate via NAD^+^ reduction to NADH. Damaged cells become leaky and release LDH into the extracellular space which then can be measured to check the extent of cell damage. Within the CytoTox 96^®^ Non-Radioactive Cytotoxicity assay, quantification of the level of LDH is done using the emission of a red formazan product that is produced in a reduction reaction involving a tetrazolium salt and NADH produced from the previous reaction. The colour intensity produced is directly proportional to the amount of LDH in the medium, which indicates the extent of cytotoxicity. 50 μL aliquots of cell media from the culture dishes post-irradiation was mixed with 50 μL of reconstituted reagent in a clear 96 well-plate, incubated for 30 min in the dark and the colorimetric reaction was measured at 490 nm using Perkin Elmer, VICTOR3™ Multilabel Counter (Model 1420). Percentage cytotoxicity was then calculated using the value of cells that were lysed using cell lysis buffer (Promega) which represented 100% cellular cytotoxicity.

### Cellular morphology

Post-irradiation morphological changes were observed after 24 hours of incubation. An inverted light microscope (Wirsam, Olympus CKX41) with a built in camera was used to capture images of the cells after treatment. 400× magnification was used to view the morphological appearance of the cells assessing the size, shape and adherence to surface of the dish. The WS1 fibroblasts were observed using 100× magnification. Extent of morphological alteration were observed and compared between the different doses. Image analysis was done using the Getit Imaging Software.

### Trypan blue cell viability

Viable cells were distinguished from non-viable cells using the trypan blue staining technique. Trypan Blue Solution, 0.4% (Invitrogen), was used to assess cell viability using the dye exclusion test. The dye exclusion test is based upon the principle that viable cells do not take up impermeable dyes due to their intact membrane, but dead cells are permeable and take up the dye. The Countess^®^ II FL (Life Sciences), an automated benchtop cell counter was used to perform cell count and viability measurements of the trypan blue stained samples. Cell viability was obtained and compared against the control group whose viability was set as the baseline.

### Adenosine triphosphate cell viability

To further determine the number of live (metabolically active) cells, after treatment, the CellTiter-Glo luminescent cell viability assay (AnaTech: Promega, PRG7571) was performed. The metabolic activity of the cells is established from the luminescent signal of a thermostable luciferase enzyme, which is proportional to the amount of ATP present in the cells. Equal volumes of suspended cells and the CellTiter-Glo reagent were added to opaque-walled 96 multi-well plate, mixed for 2 min to induce lysis and incubated at room temperature, in the dark, for 10 min to stabilize the luminescence signal. ATP luminescence was then measured on Perkin–Elmer, VICTOR3™ Multilabel Counter (model 1420). The luminescent signal is directly proportional to the amount of ATP released hence the number of viable cells.

### Statistical analysis

All sets of experiments were repeated three times (*n* = 3). Biochemical assays were performed in duplicate and an average of the results was used. Statistical analysis was performed using SigmaPlot software version 13.0 on which all calculations including the mean, standard deviation, standard error and significant changes were done. The student *t*-test was performed to determine the statistical difference between the control and experimental groups. Statistical significant difference between the untreated control and the experimental groups is shown in graphs as (*) for *p *< 0.05, (**) for *p *< 0.01, and (***) for *p * <0.001. Error bars represent the standard error on all plotted graphs.

## Abbreviations

ABC: ATP-Binding Cassette
ALDH: Aldehyde dehydrogenase
AlPcS_mix_: Sulfonated Aluminium Phthalocyanine mix
ATCC: American Type Culture Collection
ATP: Adenosine Triphosphate
Bcrp: Breast cancer resistance protein
CIN: Cervical Intraepithelial Neoplasia
CSC: Cancer Stem Cell
CD: Cluster of Differentiation
DAPI: 4′-6-diamidino-2-phenylindole
DMEM: Dulbecco’s Minimum Essential Medium
DNA: Deoxyribonucleic Acid
FBS: Foetal Bovine Serum
FITC: Fluorescein Isothiocyanate
HBSS: Hanks Balanced Salt Solution
HeLa: Henrietta Lacks
HPV: Human Papillomavirus
LASER: Light Amplification by Stimulated Emission of Radiation
LDH: Lactate Dehydrogenase
MACS: Magnetic Activated Cell Sorting
MDR: Multidrug-Resistant
PBS: Phosphate Buffered Saline
PDT: Photodynamic Therapy
PE: Phycoerythrin
PS: Photosensitizer
ROS: Reactive Oxygen Species
SP: Side Population
UV: Ultraviolet
UV-Vis: Ultraviolet-Visible.

## References

[1] Karsten U, Goletz S What makes cancer stem cell markers different? Springerplus. 2013; 2:301. 10.1186/2193-1801-2-301. 23888272 PMC3710573

[2] Marusyk A, Polyak K Tumor heterogeneity: causes and consequences. Biochim Biophys Acta. 2010; 1805:105–17. 10.1016/j.bbcan.2009.11.002. 19931353 PMC2814927

[3] Plaks V, Kong N, Werb Z The cancer stem cell niche: how essential is the niche in regulating stemness of tumor cells? Cell Stem Cell. 2015; 16:225–38. 10.1016/j.stem.2015.02.015. 25748930 PMC4355577

[4] Sell S On the stem cell origin of cancer. Am J Pathol. 2010; 176:2584–494. 10.2353/ajpath.2010.091064. 20431026 PMC2877820

[5] Borovski T, De Sousa E Melo F, Vermeulen L, Medema JP, Medema JP Cancer stem cell niche: the place to be. Cancer Res. 2011; 71:634–39. 10.1158/0008-5472.CAN-10-3220. 21266356

[6] Chhabra R Cervical cancer stem cells: opportunities and challenges. J Cancer Res Clin Oncol. 2015; 141:1889–97. 10.1007/s00432-014-1905-y. 25563493 PMC11823727

[7] Lapidot T, Sirard C, Vormoor J, Murdoch B, Hoang T, Caceres-Cortes J, Minden M, Paterson B, Caligiuri MA, Dick JE A cell initiating human acute myeloid leukaemia after transplantation into SCID mice. Nature. 1994; 367:645–48. 10.1038/367645a0. 7509044

[8] Bonnet D, Dick JE Human acute myeloid leukemia is organized as a hierarchy that originates from a primitive hematopoietic cell. Nat Med. 1997; 3:730–37. 10.1038/nm0797-730. 9212098

[9] Ortiz-Sánchez E, Santiago-López L, Cruz-Domínguez VB, Toledo-Guzmán ME, Hernández-Cueto D, Muñiz-Hernández S, Garrido E, Cantú De León D, García-Carrancá A Characterization of cervical cancer stem cell-like cells: phenotyping, stemness, and human papilloma virus co-receptor expression. Oncotarget. 2016; 7:31943–54. 10.18632/oncotarget.8218. 27008711 PMC5077987

[10] Al-Hajj M, Wicha MS, Benito-Hernandez A, Morrison SJ, Clarke MF Prospective identification of tumorigenic breast cancer cells. Proc Natl Acad Sci U S A. 2003; 100:3983–88. 10.1073/pnas.0530291100. . Erratum in: Proc Natl Acad Sci U S A. 2003 May 27;100(11):6890. 12629218 PMC153034

[11] Mor G, Alvero A The duplicitous origin of ovarian cancer. Rambam Maimonides Med J. 2013; 4:e0006. 10.5041/RMMJ.10106. 23908856 PMC3678912

[12] Schatton T, Murphy GF, Frank NY, Yamaura K, Waaga-Gasser AM, Gasser M, Zhan Q, Jordan S, Duncan LM, Weishaupt C, Fuhlbrigge RC, Kupper TS, Sayegh MH, Frank MH Identification of cells initiating human melanomas. Nature. 2008; 451:345–49. 10.1038/nature06489. 18202660 PMC3660705

[13] Singh SK, Hawkins C, Clarke ID, Squire JA, Bayani J, Hide T, Henkelman RM, Cusimano MD, Dirks PB Identification of human brain tumour initiating cells. Nature. 2004; 432:396–401. 10.1038/nature03128. 15549107

[14] Li C, Heidt DG, Dalerba P, Burant CF, Zhang L, Adsay V, Wicha M, Clarke MF, Simeone DM Identification of pancreatic cancer stem cells. Cancer Res. 2007; 67:1030–37. 10.1158/0008-5472.CAN-06-2030. 17283135

[15] Prince ME, Sivanandan R, Kaczorowski A, Wolf GT, Kaplan MJ, Dalerba P, Weissman IL, Clarke MF, Ailles LE Identification of a subpopulation of cells with cancer stem cell properties in head and neck squamous cell carcinoma. Proc Natl Acad Sci U S A. 2007; 104:973–78. 10.1073/pnas.0610117104. 17210912 PMC1783424

[16] Cui H, Zhang AJ, Chen M, Liu JJ ABC transporter inhibitors in reversing multidrug resistance to chemotherapy. Curr Drug Targets. 2015; 16:1356–71. 10.2174/1389450116666150330113506. 25901528

[17] López J, Poitevin A, Mendoza-Martínez V, Pérez-Plasencia C, García-Carrancá A Cancer-initiating cells derived from established cervical cell lines exhibit stem-cell markers and increased radioresistance. BMC Cancer. 2012; 12:48. 10.1186/1471-2407-12-48. 22284662 PMC3299592

[18] Krause M, Yaromina A, Eicheler W, Koch U, Baumann M Cancer stem cells: targets and potential biomarkers for radiotherapy. Clin Cancer Res. 2011; 17:7224–29. 10.1158/1078-0432.CCR-10-2639. 21976536

[19] Holohan C, Van Schaeybroeck S, Longley DB, Johnston PG Cancer drug resistance: an evolving paradigm. Nat Rev Cancer. 2013; 13:714–26. 10.1038/nrc3599. 24060863

[20] Doherty MR, Smigiel JM, Junk DJ, Jackson MW Cancer stem cell plasticity drives therapeutic resistance. Cancers (Basel). 2016; 8:1–13. 10.3390/cancers8010008. 26742077 PMC4728455

[21] Abrahamse H, Hamblin MR New photosensitizers for photodynamic therapy. Biochem J. 2016; 473:347–64. 10.1042/BJ20150942. 26862179 PMC4811612

[22] Mroz P, Yaroslavsky A, Kharkwal GB, Hamblin MR Cell death pathways in photodynamic therapy of cancer. Cancers (Basel). 2011; 3:2516–39. 10.3390/cancers3022516. 23914299 PMC3729395

[23] Mroz P, Hashmi JT, Huang YY, Lange N, Hamblin MR Stimulation of anti-tumor immunity by photodynamic therapy. Expert Rev Clin Immunol. 2011; 7:75–91. 10.1586/eci.10.81. 21162652 PMC3060712

[24] Dougherty TJ, Gomer CJ, Henderson BW, Jori G, Kessel D, Korbelik M, Moan J, Peng Q Photodynamic therapy. J Natl Cancer Inst. 1998; 90:889–905. 10.1093/jnci/90.12.889. 9637138 PMC4592754

[25] Calixto GM, Bernegossi J, de Freitas LM, Fontana CR, Chorilli M Nanotechnology-Based Drug Delivery Systems for Photodynamic Therapy of Cancer: A Review. Molecules. 2016; 21:342. 10.3390/molecules21030342. 26978341 PMC6274468

[26] Crous AM, Abrahamse H Lung cancer stem cells and low-intensity laser irradiation: a potential future therapy? Stem Cell Res Ther. 2013; 4:129. 10.1186/scrt340. 24153107 PMC3854767

[27] Muroya T, Kawasaki K, Suehiro Y, Kunugi T, Umayahara K, Akiya T, Iwabuchi H, Sakunaga H, Sakamoto M, Sugishita T, Tenjin Y Application of PDT for uterine cervical cancer. Diagn Ther Endosc. 1999; 5:183–90. 10.1155/DTE.5.183. 18493501 PMC2362637

[28] Barnett AA, Haller JC, Cairnduff F, Lane G, Brown SB, Roberts DJ A randomised, double-blind, placebo-controlled trial of photodynamic therapy using 5-aminolaevulinic acid for the treatment of cervical intraepithelial neoplasia. Int J Cancer. 2003; 103:829–32. 10.1002/ijc.10888. 12516106

[29] Bodner K, Bodner-Adler B, Wierrani F, Kubin A, Szölts-Szölts J, Spängler B, Grünberger W Cold-knife conization versus photodynamic therapy with topical 5-aminolevulinic acid (5-ALA) in cervical intraepithelial neoplasia (CIN) II with associated human papillomavirus infection: a comparison of preliminary results. Anticancer Res. 2003; 23:1785–88. 12820459

[30] Ramón-Gallegos E Present and future of the photodynamic therapy in cervical cancer treatment, Cervical Cancer: From Public Health to Molecular Pathogenesis. Research Signpost. 2015; c9:189–218.

[31] Hou T, Zhang W, Tong C, Kazobinka G, Huang X, Huang Y, Zhang Y Putative stem cell markers in cervical squamous cell carcinoma are correlated with poor clinical outcome. BMC Cancer. 2015; 15:785. 10.1186/s12885-015-1826-4. 26499463 PMC4619529

[32] Lee SJ, Yang A, Wu TC, Hung CF Immunotherapy for human papillomavirus-associated disease and cervical cancer: review of clinical and translational research. J Gynecol Oncol. 2016; 27:e51. 10.3802/jgo.2016.27.e51. 27329199 PMC4944018

[33] Ogunsipe A, Nyokong T Photophysical and photochemical studies of sulphonated non-transition metal phthalocyanines in aqueous and non-aqueous media. J Photochem Photobiol A Chem. 2005; 173:211–20. 10.1016/j.jphotochem.2005.03.001.

[34] Edrei R, Gottfried V, Van Lier JE, Kimel S Sulfonated phthalocyanine: photophysical properties, *in vitro* cell uptake and structure-activity relationships. J Porphyr Phthalocyanines. 1998; 2:191–99. 10.1002/(SICI)1099-1409(199805/06)2:3191::AID-JPP653.0.CO;2-4.

[35] Kresfelder TL, Cronjé MJ, Abrahamse H The effects of two metallophthalocyanines on the viability and proliferation of an esophageal cancer cell line. Photomed Laser Surg. 2009; 27:625–31. 10.1089/pho.2008.2321. 19558310

[36] Glavinas H, Krajcsi P, Cserepes J, Sarkadi B The role of ABC transporters in drug resistance, metabolism and toxicity. Curr Drug Deliv. 2004; 1:27–42. 10.2174/1567201043480036. 16305368

[37] Yang F, Gao B, Li R, Li W, Chen W, Yu Z, Zhang J Expression levels of resistant genes affect cervical cancer prognosis. Mol Med Rep. 2017; 15:2802–06. 10.3892/mmr.2017.6328. 28447725

[38] Huang R, Rofstad EK Cancer stem cells (CSCs), cervical CSCs and targeted therapies. Oncotarget. 2017; 8:35351–67. 10.18632/oncotarget.10169. 27343550 PMC5471060

[39] Aungsumart S, Vaeteewoottacharn K, Chamutpong S, Ponglikitmongkol M Chemo-radio Resistance in Cervical Cancer Induced by HPV16 E7. ScienceAsia. 2007; 33:5–11. 10.2306/scienceasia1513-1874.2007.33.005.

[40] Liu SY, Zheng PS High aldehyde dehydrogenase activity identifies cancer stem cells in human cervical cancer. Oncotarget. 2013; 4:2462–75. 10.18632/oncotarget.1578. 24318570 PMC3926841

[41] Asano T, Hirohashi Y, Torigoe T, Mariya T, Horibe R, Kuroda T, Tabuchi Y, Saijo H, Yasuda K, Mizuuchi M, Takahashi A, Asanuma H, Hasegawa T, et al Brother of the regulator of the imprinted site (BORIS) variant subfamily 6 is involved in cervical cancer stemness and can be a target of immunotherapy. Oncotarget. 2016; 7:11223–37. 10.18632/oncotarget.7165. 26849232 PMC4905468

[42] Zharkova NN, Kozlov DN, Smirnov VV, Sokolov VV, Chissov VI, Filonenko EV, Sukhin GM, Galpern MG, Vorozhtsov GN Fluorescence observations of patients in the course of photodynamic therapy of cancer with the photosensitizer photosens. Proc Internatl Symp Biomed Opt Europe. 1994; SPIE 2325. 10.1117/12.199176.

[43] Soriano J, Villanueva A, Stockert JC, Cañete M Regulated necrosis in HeLa cells induced by ZnPc photodynamic treatment: a new nuclear morphology. Int J Mol Sci. 2014; 15:22772–85. 10.3390/ijms151222772. 25501332 PMC4284736

[44] Hodgkinson N, Kruger CA, Mokwena M, Abrahamse H Cervical cancer cells (HeLa) response to photodynamic therapy using a zinc phthalocyanine photosensitizer. J Photochem Photobiol B. 2017; 177:32–38. 10.1016/j.jphotobiol.2017.10.004. 29045918

[45] Abrahamse H, Houreld NN, Muller S, Ndlovu L Fluence and wavelength of low intensity laser irradiation affect activity and proliferation of human adipose derived stem cells. MTSA. 2010; 24:8–14.

[46] Crous A, Abrahamse H Low Intensity Laser Irradiation (LILI) at 636 nm induces increased viability and proliferation in isolated lung cancer stem cells. Photomed Laser Surg. 2016; 34:525–32. 10.1089/pho.2015.3979. 26690309

[47] Chwiłkowska A, Saczko J, Modrzycka T, Marcinkowska A, Malarska A, Bielewicz J, Patalas D, Banaś T Uptake of photofrin II, a photosensitizer used in photodynamic therapy, by tumour cells *in vitro* . Acta Biochim Pol. 2003; 50:509–13. 12833175

[48] Ormond AB, Freeman HS Dye Sensitizers for Photodynamic Therapy. Materials (Basel). 2013; 6:817–40. 10.3390/ma6030817. 28809342 PMC5512801

[49] Castano AP, Demidova TN, Hamblin MR Mechanisms in photodynamic therapy: part one-photosensitizers, photochemistry and cellular localization. Photodiagnosis Photodyn Ther. 2004; 1:279–93. 10.1016/S1572-1000(05)00007-4. 25048432 PMC4108220

[50] Henderson BW, Dougherty TJ How does photodynamic therapy work? Photochem Photobiol. 1992; 55:145–57. 10.1111/j.1751-1097.1992.tb04222.x. 1603846

